# Identifying the experiences, challenges and opportunities along pathways for community-based digital skill development for young people with disabilities in Canada: a community-based research study

**DOI:** 10.3389/fresc.2026.1870194

**Published:** 2026-08-04

**Authors:** Alana Armas, Charlotte Buttle, Erica Johnston, Gabriella Avila Patro, Bliss Reynolds, Hedgerow Thompson, Darian Karuza, Ayodeji Olaomi, Kristen Rhodes

**Affiliations:** 1 Knowledge to Action, March of Dimes Canada, Toronto, ON, Canada; 2 Business+Higher Education Roundtable, Ottawa, ON, Canada; 3 Psychology Department, Queen's University, Kingston, ON, Canada; 4 Expert by Lived Experience, Kingston, ON, Canada; 5 Expert by Lived Experience, Grand Forks, BC, Canada; 6 Expert by Lived Experience, Calgary, AB, Canada; 7 Expert by Lived Experience, Toronto, ON, Canada

**Keywords:** community-based research, digital skills, employment, journey mapping, skill development pathways, young people with disabilities

## Abstract

**Introduction:**

Young people with disabilities are disadvantaged in learning and developing the skills they need to gain successful and sustained employment in Canada. This includes the development of digital skills, which are increasingly essential to participate in many aspects of society. The purpose of this study was to explore how young people with disabilities develop their digital skills outside of school.

**Methods:**

Using a community-based research approach the study specifically aimed to: 1) identify the challenges and opportunities in current community-based digital skill development pathways in Canada; and 2) develop recommendations to improve these pathways. In consultation with community members, we conducted a world café and focus group, which then informed persona development, journey mapping, and recommendation ideation exercises. A total of 22 participants were included in the study.

**Results:**

Our study developed seven pathways that captured the diverse experiences and complex ways young people with disabilities attain digital skills in Canada. The pathways revealed that many young people seeking to improve their digital skills need to start with information seeking, balance growth while managing their disability, continually take initiative, maintain self-motivation and self-advocacy, and cope with emotional stress. We also found that cost, inaccessible programs and technology, rapid technological change, fragmented systems, andwork place inequity are ongoing challenges to digital skill development, while community support and free online resources create key opportunities for young people.

**Discussion:**

Through community consultation, we concluded that accessible and inclusive community-based digital skill development pathways require systemic change in Canada, led by the federal government, supported by collaboration across systems and organizations, and anchored in the lived experiences of young people with disabilities.

## 1 Introduction

Across Canada, the number of people living with one or more disabilities has steadily increased, growing from 22% in 2017 to 27% in 2022 (1). With at least one in four Canadians experiencing disability, there is a growing recognition that people with disabilities face numerous barriers that ultimately lead to poorer health and quality of life outcomes (2, 3). It is well known that people with disabilities often encounter barriers in their everyday lives, such as financial limitations, transportation and housing barriers, as well as stigma and stereotypes (2, 3). These barriers can negatively impact multiple aspects of their lives, including making it difficult to engage in healthcare and healthy lifestyle practices, increasing food and housing insecurity, exacerbating social isolation, limiting opportunities to access education, and making it difficult to secure and maintain employment. To begin addressing these compounding barriers there has been an increased focus on bettering Canada's systems, institutions, and government policies to improve the lives of people with disabilities. In 2019 the Canadian government passed the Accessible Canada Act, aiming to make Canada barrier-free by 2040. Since then, millions of dollars have been invested into better understanding the experiences of Canadians with disabilities, removing barriers, and developing solutions.

To improve the lives of people with disabilities, many groups across Canada approach addressing barriers through the well-established social model of disability (4–6). While not ubiquitous, the lens of many non-profit and public sector organizations is to address disabling barriers in social and built environments, or build new fully accessible environments, rather than focusing on individual impairments (4). One area where the government and organizations have applied this thinking has been working to improve is creating opportunities for people with disabilities to secure and maintain employment (7), as evidence shows hiring people with disabilities can positively impact employers and the workforce (8). There is also ample evidence that sustained and secure employment is a social determinant of health (9–11), and creating more opportunities for employment could address some of the barriers people with disabilities face. Between 2016 and 2021 there was a slight increase in the employment rate for working age Canadians with disabilities from 59% to 62% (1). However, even with this increase, there remains a 16-percentage point difference between the employment rate of working-age adults with disabilities and those without disabilities, which stands at 78% (7). Further, evidence shows that people with disabilities are often in more precarious employment situations, working part-time or casual jobs, and the wage gap between people with disabilities and those without disabilities in Canada has widened to a difference of $2.22 in 2024 (1, 12, 13).

In particular, young people with disabilities (YPWD) face significant challenges in securing employment in Canada, including a nearly 7% decline in the employment rate among those aged 15–24 with disabilities in 2024 (12). For many people, the period of life between ages 15 and 30 is filled with transitions, from finishing secondary school, entering post-secondary education, seeking full-time employment, and focusing on career advancement (14). Research has shown that when young people face challenges with employment early in their career it can create long-term negative impacts that can extend into adulthood (14), such as reduced earnings, lower quality jobs, slower career progression, and reduced financial stability over time (14, 15). The risk of these long-term negative impacts is higher for young Canadians as they face employment challenges caused by more people entering the workforce, fewer full-time positions, increasing education costs, and fewer career development opportunities (16).

These are significant challenges, and evidence shows that YPWD are disproportionately impacted (16, 17), resulting in YPWD having the lowest employment rate compared to other marginalized groups in Canada (16). Research has shown that YPWD between the ages of 15 and 19 worried about isolation by co-workers (18, 19) and thought that employers would not want to hire them because of their disability. Stigma can also cause YPWD to struggle with labelling their disabilities and deciding whether or not to disclose that to their employers (18, 19). These worries are reflected in the evidence where it has been found that young people with moderate to severe disabilities report more discrimination in employment including being refused an interview, promotion, job accommodation, and/or given less responsibility (20). Last, hiring processes such as inaccessible online platforms and job applications, the use of AI for screening applications, and standard assessment practices (21) can all create additional barriers to employment for YPWD.

Addressing employment barriers is complex and will require a multi-faceted approach to improve the lives of YPWD. With the rapidly changing labor market in Canada, one approach to supporting YPWD is to remove barriers to skill development. Evidence shows that YPWD make up one third of youth aged 15–29 who are not employed nor engaged in education or training (NEET) (16), suggesting that creating more accessible opportunities for YPWD to develop skills could help them build the foundation they need to secure employment. Specifically, creating more opportunities to develop digital skills is imperative for YPWD. As Canada's economy continues to experience a digital transformation, possessing digital skills (e.g., computer literacy, office productivity, e-communication, cybersecurity) is necessary to obtain and maintain competitive roles. Moreover, Canadian employers are seeking workers who have advanced digital skills that go beyond basic skills like being able to interact with a computer, use word processing software, or engage in communication software (e.g., Zoom, MS Teams) (22).

YPWD are disadvantaged in learning these skills because of systemic inequities such as a lack of infrastructure in the education systems and the availability of digital technologies (23). For example, in 2024, 45% of Canadians with disabilities or long-term conditions reported condition-related barriers to their online activities, and 57% faced barriers when using information and communication technologies (24). In school, research has found that young people are often placed in specialized classrooms with fewer opportunities to access computer skills training (25), putting them at a distinct disadvantage for digital skill development. Compounding this is the digital inequity YPWD often face outside of school, where they are half as likely to own computers and a quarter as likely to use the internet (26).

As all aspects of employment increasingly require people to use technology, including online applications and virtual interviews, the near ubiquitous use of digital platforms for word processing and data entry, as well as scheduling applications and online payroll platforms, it is imperative that YPWD have the skills required to participate in these environments. Digital skills will also become even more important with the rise of AI, where workers are increasingly expected to understand how to integrate tools like generative AI into their workflows and use new skills like prompt design. While education systems have been teaching digital skills and using technology in classrooms for many years, YPWD still face barriers. Limited to no infrastructure and little availability of accessible technologies compounded with teaching staff that lack knowledge about accessible technology (23) can make it challenging for students with disabilities to improve their digital literacy and learn digital skills. To address this gap in the formal education system, there is some research on how community-based interventions can support digital skill development (27–30), as well as many community-based programs supporting employment in Canada that are entirely or partly focused on digital skill development. This is promising, yet barriers to these programs and digital skill development for YPWD, such as cost, inaccessible platforms, and limited access to technology persist. To create more opportunities for YPWD to access digital skill development in Canada there is a need to better understand current pathways that exist outside of the education system. Being able to determine the challenges and strengths in current pathways can help decision makers take steps to remove barriers. Current research looks generally at access barriers for YPWD but there is limited to no research looking specifically at the pathways that lead to digital skill development.

Understanding pathways to skill development, specifically for digital skills, in Canada could help identify where and how we can address current barriers. In turn, creating better pathways to digital skill development could increase YPWD's employability, leading to better financial security, more opportunities for full-time employment and further career development. Improved access to digital skill development could also help YPWD who may be unable to work to better access health care, social networks, and other supports they need in their daily living. Thus, pivoting research to better understand how to enable YPWD to engage in our digital society and economy is essential. This study is grounded in the social model of disability (4–6), aiming to explore the challenges and opportunities of community-based pathways to digital skill development in order to remove barriers or uncover new ways to build a social and built environment that does not limit YPWD. Our study had two main objectives: 1) determine what current digital skill development pathways look like for young people with disabilities in Canada; and 2) identify how current pathways can be improved to remove barriers and create more opportunities for YPWD.

## 2 Materials and methods

### 2.1 Design and setting

The study used a community-based research approach, where community members and researchers worked closely together to ground the research in community needs and interests. The study followed six core principles for community-based research (31, 32), ensuring that throughout the study all activities were: 1) participatory; 2) cooperative; 3) promoting a co-learning process; 4) employing methods for systems development and local community capacity building; 5) empowering to community members; and 6) balancing research and action. The study was conducted at March of Dimes Canada, a national community-based organization in Canada that provides a variety of services and supports to people with disabilities. The study took place from November 2024 to December 2025.

To support our community-based research approach, we established three groups of stakeholders to advise and participate in the study. The first group included 7 young people with disabilities (YPWD) who we met with monthly; the second group included 7 people from other community-based disability organizations and subject matter experts in employment, disability, inclusive practices, and skill development we who met with every three months; and the third group included 13 staff from March of Dimes Canada who had experience working with YPWD and/or providing employment and digital skill development support who we met with every three months. During our meetings with these groups we would discuss methods, early insights, identify opportunities for knowledge sharing, validate the final study results, and generate a set of recommendations to action the research findings. Meeting with these groups throughout each phase of the study ensured our approach, findings, and recommendations remained grounded in community perspectives and needs, while also identifying opportunities to action the findings of the study.

We received ethics approval from the Community Research Ethics Office to conduct this study; the approval number is #435.

### 2.2 Participants

The study was primarily focused on young people with disabilities. Specifically, we were interested in including participants who were: 18–30 years old; currently living anywhere in Canada; and identified as a person with a disability.

We selected this age range to represent young people because there is no set consensus on what age ranges fall within the concept of ‘young people', generally it tends to be defined between the ages of 15–24 (33) or 18–25 (34). Based on discussions with people with disabilities, it was important for us to acknowledge that some YPWD engage in transitions such as completing school, moving out of their parents' or caregivers' homes, and becoming employed at a later age than their counterparts. Therefore, it was agreed that increasing the age range of young people to 30 years old was appropriate, which is also supported by the literature (34).

Instead of focusing on specific disabilities, we chose to only require participants to identify as a person with a disability. We did this because what defines disability is an ongoing, evolving and complex conversation. The way disability is understood has changed drastically over time, and it will likely continue to change. So, for the purposes of this study we did not take a disability specific approach to recruitment.

We also recruited professionals from community-based organizations to participate in the world café and focus group bringing additional perspectives and experiences to the data. Specifically, we were interested in recruiting participants who: currently live and work in Canada; have experience working with YPWD; and have experience providing employment and/or skill development support to people with disabilities. For both YPWD and professionals we used a purposive sampling technique.

### 2.3 Phase one: world café and focus group

The first phase of the study included a world café and focus group to better understand current challenges and opportunities that young people with disabilities in Canada face when accessing digital skill development programs, services, and materials.

#### 2.3.1 World café

We conducted a world café with YPWD and professionals from community-based organizations to understand the challenges and opportunities YPWD encounter when looking to improve their digital skills. A total of 10 participants attended the event, 7 YPWD and 3 community and sector experts. A world café approach (35, 36) is a qualitative method where a large group of participants are split into smaller groups then rotate through a series of tables discussing specific topics, building off the discussion of previous groups in order to strengthen and refine insights. This approach was ideal for this study so we could facilitate smaller group discussions covering three topics (digital skills, non-traditional skill development, and tech-enabled jobs), allowing all participants to contribute to the topics and questions. The world café format also added more richness to the data since participants built on previous groups' reflections.

The event was three hours in length and took place via Zoom to allow for participation from across Canada. Participants were split into groups that rotated through three breakout rooms, each room covering one of the topics listed above. After all groups completed the breakout rooms, we brought all the participants back together to review and discuss the key takeaways from each discussion.

We used the framework approach (37) to thematically analyze the transcripts from each breakout room and the general discussion. The framework approach is a structured approach to qualitative data analysis that allows researchers to thoroughly identify patterns and themes while maintaining a clear audit trail, making it useful when multiple researchers are analyzing qualitative data (37). AA, CB, and BR independently created the *in-vivo* codes, preliminary thoughts, and resulting initial categories for each breakout room transcript, then reviewed and validated each other's initial categories. BR also completed these steps on the general discussion at the end of the world café. Once this was completed, we held several analysis sessions where we brought together all the initial categories to generate the initial themes, which we then used to refine categories, identify the final themes and build the overarching core concepts.

#### 2.3.2 Focus group

To complement the world café event, we conducted a 60-minute focus group with five coordinators and providers from a program that offers skills training and employment opportunities for high school students with disabilities. The program does not specifically focus on digital skills, instead offering general skills training that include digital skills. The purpose of the focus group was for the program staff to share their observations on the digital skill level of high school students who went through the program, the challenges students face, gaps in expectations between students and employers, and how the program supports students.

We also used the framework approach to thematically analyze the focus group data.

### 2.4 Phase two: pathway development and analysis

The second phase of the study focused on creating pathways of digital skill development that are informed by lived experience, evidence, and the findings from phase one. To complete this work, we developed personas, then conducted journey mapping exercises, and finally completed a gap analysis to identify recommendations on improving these pathways.

Since the aim of the study was to better understand community-based digital skill development pathways for YPWD it was essential to account for the diversity and intersectionality within the disability community. For this reason, we employed a UX-inspired process, first developing detailed personas to inform the journey mapping exercises. Jacob et al. (38) states that personas can offer more efficient context-rich communication in the design process, helping move away from thinking of an abstract user and instead centering the user during design. Moreover, Ward (39) states that personas help to more clearly see users' needs when designing services, making the initial step of persona creation appropriate for this phase of the study. Once the personas were developed, we used Endmann and Kebner's journey mapping process (40) to develop the pathways. Their process aimed to learn in a short time user needs and identify areas for further research (40), making it an ideal approach for this study. The structured approach allowed us to generate several pathways that reflect the diverse ways YPWD develop their digital skills, while also capturing the diversity and intersectionality within this community.

#### 2.4.1 Persona design

To create the personas, we held two sessions with seven YPWD. In these sessions we developed a digital skill spectrum made of seven different skill levels, ranging from no experience to expert proficiency, to form the basis of the personas. These personas represented a diverse range of disabilities, including neurodivergence, physical, sensory, and mental health conditions. The personas were also designed to capture a range of intersecting identities and experiences. To ensure the personas were grounded in both lived experience and evidence, the research team used academic literature, community evidence, and population data to validate the personas' characteristics. These persona profiles were then validated with the third stakeholder group for the project, which comprised community-facing and service-providing staff members at March of Dimes Canada. In this session, we assessed the alignment of the personas with real-world client profiles based on their service delivery experience. Feedback was provided to improve representativeness and completeness. For example, revisions included incorporating pathways related to acquiring a disability through workplace injury and representing individuals living in low-resource settings.

#### 2.4.2 Journey mapping

**Figure 1:**
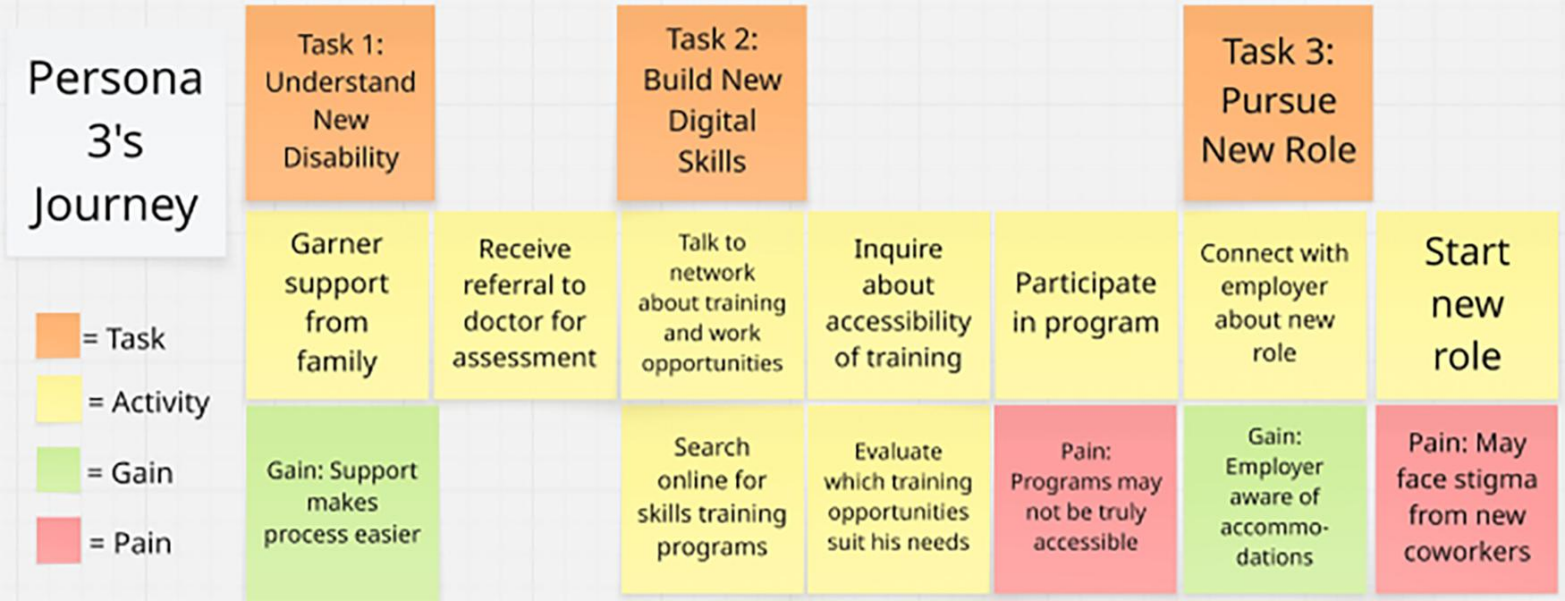
Simplified example of journey mapping exercises.

To develop the pathways, we conducted three sessions to complete the journey mapping exercise. In accordance with Endmann and Kebner's (40) approach, in the first session we identified the triggering event and end goal for each of the validated personas. This was informed by the individual motivations, backstory, and characteristics generated for each persona. Once a triggering event and end goal was agreed upon, the YPWD brainstormed all potential activities that would need to occur for that persona to achieve their end goal. With the comprehensive list of activities generated, the next session focused on reaching a consensus on which activities formed broader tasks. These tasks then formed the basis of the pathways. In the final session, we reviewed the pathways and captured areas the YPWD identified as pain points, gains, emotional experiences, and potential recommendations for increasing YPWD's success and reducing barriers along the pathways. Figure 1 demonstrates the mapping process. Once this work was completed, the YPWD reviewed summaries for each pathway that included the persona, the triggering event, end goal, generated activities, tasks, pain points, gains, emotions, and recommendations. The YPWD were then asked to suggest changes or additions, and validate the pathways.

#### 2.4.3 Recommendation ideation

To complete this phase of the study, we held a final session where we led the YPWD through two exercises called “painkillers and vitamins” (41) and “magic wanding” (42). For the “painkillers and vitamins” exercise, participants were presented with a digital whiteboard containing all the pain points that emerged along the pathways during the journey mapping process. These pain points were loosely grouped together thematically prior to the session to allow for the generation of ideas that may address cross-cutting pain points that emerged across multiple pathways. To ground the discussion “painkillers and vitamins” were defined to participants as “actionable strategies . to address identified challenges (painkillers) and enhance enablers (vitamins)” (41). For the “magic wanding” exercise, YPWD were “encouraged to think beyond existing limitations and imagine ideal solutions” (42). In this exercise we presented the persona profiles and a description of the initiating event and goal for each pathway and asked participants to describe what a world looks like where this persona thrives digitally. With these two ideation methods, we identified both practical solutions and aspirational ideas to improve community-based digital skill development. The data from these sessions were then synthesized to form potential recommendations.

#### 2.4.4 Gap analysis

To synthesize insights generated from the persona design, journey mapping, and recommendation ideation activities, CB and EJ conducted a thematic analysis of all identified pain points, gains, and recommendations. Pain points were first coded and grouped into thematic categories to identify cross-cutting barriers and overarching themes that appeared across multiple pathways, highlighting gaps and commonalities in the current-state experience. Using the same approach, gains were coded and organized into thematic categories to surface common facilitators and positive experiences. Finally, recommendations generated during ideation sessions with the YPWD stakeholder group where ideas were clustered based on conceptual similarity and mapped to the previously identified gaps (i.e., the cross-cutting pain points) to illustrate how proposed ideas addressed specific unmet needs. The recommendations were then validated with the stakeholder groups comprised of community and sector experts, and MODC staff. These groups reviewed the outputs and provided structured feedback on their feasibility, alignment with funder jurisdictional priorities, and completeness.

The refined recommendations were then reviewed again by all three stakeholder groups to finalize a consolidated set of recommendations. This iterative, multi-stakeholder validation process ensured that study outputs were grounded in both lived experience and service delivery perspectives.

## 3 Results

The study findings revealed complex pathways, challenges, and opportunities that YPWD face when looking to develop their digital skills no matter how basic or advanced their digital skills may be, see Table 1. The pathways developed for the seven personas captured the complexity of living with disability in Canada, and how that shapes an individual's experience of improving their digital skills. The section below provides a narrative summary of the seven pathways that were generated with YPWD, informed by findings from the world café and focus group, and validated with evidence.

**Table 1:** Summary of pathways.

Digital Skill Level	None	Low	Basic	Moderate
**Persona**	Abigail	Jessica	Evan	Mvondo
**Age**	18	20	22	27
**Disability Type(s)**	Autism, ADHD	Pain-Related, mental health	Traumatic brain injury, depression	Spinal cord injury
**Gender Identity**	Woman	Woman	Man	Non-binary
**Ethnicity**	Mennonite	Indigenous	White	Black
**Education Level**	Completing high school diploma	Less than high school diploma	Trade apprenticeship	Bachelor's degree
**Employment Status**	Part-time employment	Unemployed	Unemployed	Full-time employment
**Location**	Linden, AB	Winnipeg, MB	Parry Sound, ON	Montreal, QC

Digital Skill Level	Intermediate	Advanced	Expert	
**Persona**	Khalid	Shannon	Satvinder	
**Age**	29	25	31	
**Disability Type(s)**	Dyslexia	Cerebral palsy	Low vision condition	
**Gender Identity**	Man	Woman	Man	
**Ethnicity**	Mixed background	White	South Asian	
**Education Level**	College diploma	Master's degree	Master's degree	
**Employment Status**	Unemployed	Full-time employment	Full-time employment	
**Location**	Mississauga, ON	Saint John, NB	Vancouver, BC	

### 3.1 Pathways to digital skill development in Canada

#### 3.1.1 No digital skills: Abigail

Abigail, an 18-year-old Mennonite woman, high school student, with autism and ADHD, from Linden, Alberta is considering her future and is inspired to become more independent by improving her digital skills. She would like to feel more confident working at her family's business (a grocery store) and be able to conduct online research to learn more about her disability and possible accommodations.

To begin her journey, she first learns digital research skills through friends, community programs, and workshops. She then uses these new research skills to discover potential routes where she can learn more digital skills and incorporate accessible technology into her work.

From her research, she also selects the technology she thinks will work best for her needs and then learns how to use it through generic training before pursuing more tailored learning through online platforms like Reddit and through disability organizations. However, her high-school curriculum does not teach her how to use this technology, requiring her to seek external resources. Abigail finds that the first generic training she attempts to learn from is not accessible for her. After trial and error, Abigail notices that the self-paced learning of online courses offered through community organizations is helpful for learning with her ADHD.

#### 3.1.2 Low digital skills: Jessica

Jessica, a 20-year-old Indigenous woman from Winnipeg, Manitoba, is unemployed without a high school diploma and has experienced being unhoused. She is in the process of applying for provincial disability support to help with her expenses related to comorbid pain and mental health disabilities. She has recently secured social housing, providing her more stability and opportunity for independence. As a result, she feels ready to complete high school and seek employment.

In her journey, she seeks out opportunities to complete high school online. However, Jessica is also concurrently managing her goals of education and employment as well as managing her disability, trying to get approved for provincial disability support, and trying to connect with healthcare professionals and social workers. This comes with a host of bureaucratic challenges like navigating eligibility requirements, completing paperwork, obtaining sufficient medical documentation, and waiting for responses.

The majority of her activities, like researching her options and submitting forms online, have barriers Jessica must first overcome. Jessica's activities require that she has a baseline level of digital literacy as well as physical and financial access to devices and the internet. To overcome some of these challenges, she uses what she has access to, like the local library, YouTube, and peers, to help her develop the skills she needs to complete her tasks. Early acquisition of digital skills is critical in Jessica's pathway, such as digital research, online learning platforms, virtual classrooms, and productivity tools. She can then use her newfound skills to search for programs to further advance her digital skills, to complete the requirements for her high school diploma, and to seek opportunities to build her employability (e.g., employment skills programs, learning how to make a proper resume).

#### 3.1.3 Basic digital skills: Evan

Evan is a 22 year-old white man from Parry Sound, Ontario, who sustained a traumatic brain injury and developed depression following a workplace accident. He holds a trade school diploma and worked as a boiler mechanic but is currently unemployed due to his acquired disability. Evan seeks to transition into a new position that is better suited to his needs. Since his accident has led to substantial changes in his life, garnering social and emotional support from his network as well as professionals is an essential first step.

To start his transition into a new role, his old workplace facilitates his connection with healthcare professionals to assess the state of his skills. Evan works with various rehabilitation services and an occupational therapist until he is cleared to return to work.

While physically ready to return to work, he now has to work on digital upskilling to prepare for the more administrative tasks he will be responsible for in his new role. He begins to research potential programs but has a lot to consider while doing so, such as accessibility needs, his eligibility for certain programs, and navigating the costs his employer is willing to cover for his training. Upon figuring out these details, he enrolls in multiple courses, but to fully participate he still has to work through ensuring he has the appropriate technology and software to complete the coursework. Luckily, he has his family supporting him through every step.

Evan then feels ready to return to work. Throughout his journey of navigating emotional support, medical treatment, and finding accessible programs, Evan has become adept at advocating for his needs. He connects with his company's human resources with a cover letter and resume highlighting his new digital skills to initiate the process. He completes some interviews and asks for the accommodations he needs. After his hard work, he begins his new role, complete with accessible productivity tools and a plan in place for his employer to provide continuing support depending on his needs. While having to maintain reporting to his employer and community agencies regarding his needs and progress, Evan is successful in his new job, with his family and newfound disability community cheering him on.

#### 3.1.4 Moderate digital skills: Mvondo

Mvondo is a 27-year-old non-binary Black immigrant working as an education assistant in Montreal, Quebec. They are a French speaker who uses a wheelchair following a spinal cord injury. At work, Mvondo meets a student who has accessibility needs that they do not have the knowledge and tools to support. As a dedicated educator, Mvondo decides to learn more about assistive technology in order to support all their students.

Unfortunately, the professional development processes in their workplace fail to provide timely, structured support. To achieve their goal, Mvondo spends significant off-hours researching student disability specific needs and accessibility tools. They also connect with their colleagues and students to learn from their experiences. Lack of preparedness shifts responsibility onto students to educate staff, creating inequity and stress for both parties.

Mvondo takes what they learned from their research and tries through trial and error to apply it inside the classroom. This is, however, insufficient, and there are still gaps in what the students need and what they can provide. They then pursue certifications, through organizations like the Rehabilitation Engineering and Assistive Technology Society of North America, and seek other specialized training outside employer support. To access these opportunities Mvondo has to pay out-of-pocket because school board funding is slow or unavailable.

#### 3.1.5 Intermediate digital skills: Khalid

Khalid, a 29-year-old man, graphic design graduate with dyslexia, is an eager graphic designer seeking employment. He lives in Mississauga, Ontario and is from a mixed Middle Eastern and Latin American heritage. He is very involved in his community, dedicating time to volunteering and teaching others about technology. He often also finds himself providing technological support to his older family members. Though he is well-versed in technology and graphic design, technology is always advancing. One day while browsing graphic design openings on a job board, Khalid comes across a skill requirement for a position that he is unfamiliar with. He quickly takes action exploring the current job market to take inventory of what skills he has and is missing.

He decides to pursue additional training for the missing skills, starting with broad research and tips from others' experiences on online communities like Reddit and Discord. This information is helpful, but he needs support learning how to execute these particular skills accessibly. Khalid first connects with disability organizations to support him with general training on incorporating assistive technology into his graphic design skills, but this does not meet his full needs. Advanced digital skill courses tailored for people with disabilities are scarce. He finds additional courses and training more specific to the skills he is looking to develop, but since they lack accessibility options, Khalid must navigate disclosure strategies and accommodation requests.

Khalid successfully refines his graphic design skills but is worried about his chances of securing the job he wants as a person with a disability. He knows he has a strong portfolio, but he worries that employers may perceive his disability as a limitation. In response to these feelings Khalid seeks out job readiness courses and specific support for people with disabilities on navigating disclosure and accommodations in job applications. Upon completing these training courses, Khalid begins applying and interviewing for graphic design positions. He still has fears about stigma and misconceptions but is able to use the tools he learned to disclose his disability and request accommodations from employers.

#### 3.1.6 Advanced digital skills: Shannon

Shannon is a 25-year-old woman, IT specialist working at a university in Saint John, New Brunswick. She has cerebral palsy. Shannon is great at her job but feels pressure to stay competitive among the advancing tech-world. She has the idea to build an AI-powered FAQ bot for her workplace. Formal education and workplace training lag behind industry trends, so Shannon has not received any training on AI. She begins this project by conducting comprehensive research online about AI, how others have built similar programs, and what tools are available to help her. Since AI is a hot topic, there is a flood of new resources, many of which are unvetted or of low quality that Shannon must navigate.

Feeling more informed, she writes a proposal for her employer to fund this project. Upon approval, which took many months, she enrolls in general learning courses about AI and then completes specific training on the software she decides to use for the project. She practices her AI skills until she feels confident to create a demo of her chat-bot. She does user testing and feedback with the demo and then works on creating the final version that is then launched university wide.

#### 3.1.7 Expert digital skills: Satvinder

Satvinder is a 31-year-old South Asian man from Vancouver, British Columbia. He is the CEO of his own tech company. He was recently diagnosed with a degenerative low-vision condition. His symptoms had been progressing for a while, and he was forced to confront them realizing he was struggling to work. After keeping his vision challenges to himself for a long time, he finally tells his family of his diagnosis so they can support him in his journey to adjust to his developing condition. Not wanting to leave his company, Satvinder decides he will learn about the accessible technology and assistive devices needed to continue working.

From his doctor, he is referred to Vision Loss Rehabilitation Canada and Canadian National Institute for the Blind, who are very helpful in introducing him to various assistive technology items and helping him learn how he can use the technology at work. Satvinder's job demands expert technological knowledge and skills, and the tools provided by community organizations do not solve all his needs. The mainstream technological tools and software used at his company lack built-in accessibility features. Luckily, due to his advanced skills and economic status, he hires private specialists and conducts independent research on how to complete all aspects of his life accessibly. He is also able to purchase all the new, top-of-the-line assistive technology. While Satvinder can afford private tutors and equipment, this cost burden would be prohibitive for most.

### 3.2 Defining moments when improving digital skills

When seeking to attain or improve digital skills through community and self-directed means, we found several common defining moments YPWD experience across the varying goals and circumstances. These moments point to systemic patterns and shared challenges, including 1) starting with information-seeking; 2) balancing growth while managing their disability; 3) the need for initiative, self-motivation and advocacy; and 4) coping with emotional stress.

#### 3.2.1 Starting with information-seeking

When looking to improve skills outside of the education system, the pathways highlighted that YPWD need to independently search for resources and services. This research can come in different forms. In Khalid's journey, he engaged with online forums like Reddit and Discord to find ways for improving his digital skills. Jessica's journey also highlights that even when trying to re-enter the education system to complete her high school diploma there is a level of searching that is required, which mostly takes place online. We also see this too with Abigail, who is seeking additional accessible tools and information that is not available at her high school. This initial step on every pathway is consistent with what we heard from participants in the world café, where finding solutions online was normal and expected:

“[there is] a lot of the self-teaching, elementary school and high school didn't help too much. It's more with friends and sort of absorbing that information and passing it along.” (World café, YPWD).

It is important to highlight that the pathways show that regardless of motivation or skill level, individuals must navigate complex information ecosystems before taking meaningful steps towards their goals. The reliance on digital searching underscores the critical role of digital literacy and points to additional barriers for those with limited digital skills. Yet, even with the need for baseline digital skills, study participants reflected that accessing resources and programs in the community is often easier than in the education system, and can sometimes even offer support that does not exist in the education system.

#### 3.2.2 Balancing growth while managing their disability

Across pathways there is a recurring theme of the tension between pursuing growth opportunities and managing the realities of living with a disability. Many pathways recognize the existence of coexisting and codependent priorities for the personas. For example, in Jessica's journey mental health supports and accessing disability funding added additional complexities in navigating her pathways, even if they were not the instigating factors to her desire to complete high school. In many instances, the pathways also demonstrate the compounded time and effort burden brought on by navigating ableist environments just to reach parity with their non-disabled peers. World café participants reflected how this tension is present when seeking employment, where sustained employment was seen as a fundamental pre-requisite for being in good health, yet poor health or disability was also described as being a large barrier to seeking employment, creating a contradiction that is difficult or sometimes impossible to navigate. In many cases, participants reflected that individuals needed to be able to manage the complexity of living with a disability in order to pursue personal or professional goals.

#### 3.2.3 Initiative, self-motivation, and advocacy

The pathways demonstrated the need for YPWD to have initiative and perseverance to successfully build their skills. Consistent with the everyday experience of having a disability, the pathways revealed that YPWD need to navigate skill development while simultaneously requesting accommodations and support, working within systems and environments where accessibility is not a priority, and consistently advocate for themselves. We see this most acutely in Mvondo's journey where they were aiming to learn more about accessible technologies and spent a significant amount of time working within systems where accessibility was not a priority.

Another key finding that emerged from these pathways was limited formal support. The pathways highlighted the complexity of building skills outside of the education system, revealing that self-directed learning and connections are essential. We see this in Khalid's journey where he depended on the online community, or with Evan where support from his family helped him succeed in his work transition. These pathways reflected the experiences of participants across the project, where they often spoke of their own experiences advocating for themselves, or relying on their families and friends to help them learn new skills or find resources and programs. It is important to note that one participant shared while support from family and friends can be helpful along these pathways, living with a disability can be isolating and having a network of family and friends may not be typical.

#### 3.2.4 Coping with emotional stress

An important theme across all pathways was the emotional toll of navigating digital skill development. In creating these pathways with YPWD we were able to capture the complexities of community-based skill development as well as how this impacts individuals. Throughout the pathways we consistently saw motivation and excitement accompany initial steps, while burnout, worry, and grief emerge during systemic delays or skill acquisition challenges. Several personas showcased feelings of pressure, stigma, or inequity as they navigated inaccessible systems, such as Khalid being worried about employer bias during his job search, or Mvondo feeling stress from inadequate workplace preparedness, and finally Satvinder who grappled with the reality of his diagnosis. We also found that support networks such as family, colleagues, and community organizations played a critical role in helping individuals cope and maintain resilience throughout their journeys.

### 3.3 Challenges and ‘pain points'

Several challenges and ‘pain points' emerged from the findings, highlighting areas where YPWD are experiencing difficulties navigating community pathways to skill development. We found that multiple intersecting and compounding health, social, economic and environmental factors shape YPWD's ability to build skills from 1) the costs of pursuing community-based skill development; 2) the lack of accessible and universal design; 3) the rapid evolution of technology; 4) the shortcomings of formal systems; and 5) structural inequities in career progression. All of these challenges created ‘pain points' throughout the pathways, highlighting where current systems are creating barriers for YPWD.

#### 3.3.1 The costs of pursuing community-based pathways

Participants across the study were clear that while community-based pathways to digital skill development offered greater flexibility and sometimes greater access to the skills YPWD needed for employment, there were often financial costs that created barriers. First, participants spoke of the financial costs associated with skill development that is present both in community-based and formal educational programs. Most obvious was the reflection that not all community-based programs are free, that courses offered via LinkedIn or Coursera can have fees. This is particularly true for courses aimed at acquiring more advanced digital skills. The study found that in Canada community and government organizations offer free resources and programs for digital skills, but these tend to focus on basic digital skills. Participants noted that even within these programs there can be costs for purchasing technology or accessibility features so they can even attend the course. Compounding this, community-based programs can have limited availability or strict funding requirements, so there can be high levels of bureaucracy YPWD must navigate to attend. Individuals often have to meet certain requirements or submit applications to be approved for programs and services they need, and then often go through long wait times to be approved and able to attend.

Accessing free online resources can offer an alternative path for YPWD, with participants reflecting on being self-taught and learning from platforms like YouTube. One participant shares that YouTube can offer “extra… reinforcement… for concepts that you might not have learned very easily, you know, in a more traditional setting.” (World café, YPWD) We also saw this come through in the pathways where personas with lower incomes, like Abigail and Jessica, were often seeking free resources offered online. While this may be a good alternative, it is not without it challenges, such as needing to navigate misinformation or scams, or finding free resources that are limited or basic in their teachings, lacking the depth needed for advanced skills. As we saw in the pathways, it was with the personas who had access to greater financial supports, which may not be typical for YPWD, where learning more advanced digital skills was available. This is seen with Shannon's journey where her employer funded her skill refinement, and Satvinder's journey where he was able to pay for training.

#### 3.3.2 Accessibility is often an after thought

One key finding that served as a critical barrier to digital skill development is that accessibility is often a secondary consideration, or not considered at all. There are several instances of participants sharing how programs or technology were not created with accessibility in mind, creating barriers, and requiring additional advocacy. One participant shared:

“I was then able to take an IT course [that] was more or less a course for everyone. It's not limited to people that are visually impaired. You had to do some advocacy for yourself and negotiate recommendations here and there because not everything is accessible in the IT space.” (World café, YPWD).

When searching for options to learn digital skills or obtain certifications, programs for the general population tend to be most popular and are thus found first. However, as the participant reflected, many of the most available options often do not have accessible use options built into them. This was reflected in the pathways where, for example, Abigail first enrolls in a more popular, generic digital skills course. This course turns out to be inaccessible for her, causing her to find a different course better suited to her accessibility needs.

We also found that even when participants were able to get accommodations approved and implemented, they reflected that the length of the accommodation process often put them at a disadvantage.

“We know getting an accommodation is not a one-day thing. It takes sometimes weeks to finalize what those are. If it's a fast-paced training you might already be behind. … If you're not able to catch up, sometimes you might have to leave the training program.” (World café, YPWD).

Satvinder's journey also reflects the lack of focus on inclusion and accessible technologies. His journey demonstrated that while independence and financial stability can address barriers, accessibility is rarely a priority or easily achieved.

Building on this, YPWD may also encounter technology that is inaccessible. Each time YPWD use new hardware or software they may need to take time to figure out how to use the accessibility features as it is often not straightforward. One participant reflected on this saying:.

“I didn't know how to use screen readers on Windows. I could only use screen readers on Android. So that was very eye opening because I learned a lot of stuff just in the Windows OS.” (World café, YPWD).

Khalid's journey reflects this experience, where he is actively trying to remain a competitive candidate for graphic design jobs by educating himself on new skills and keeping up to date with design trends, while also navigating his accessibility needs. Khalid's journey and the participant's reflection demonstrate that when accessibility is a secondary consideration it can be extra challenging for YPWD, where they may need to consider how to learn a new skill in a way that support their individual needs.

#### 3.3.3 The ubiquitous use and rapid evolution of technology

A prominent finding is the unique challenge that technology creates for YPWD when developing digital skills. Participants observed that technology is ubiquitous in everyday life, requiring everyone to have some baseline digital skills. Many participants reflected that the COVID-19 pandemic accelerated the uptake of virtual and digital technologies in all aspects of life.

“COVID sped everything up, we have to learn digital skills to be able to survive in everyday life. Whether we're paying our rent or our mortgage, whether we're paying our bills online. Whether we're sometimes shopping, it's easier done utilizing apps and delivery services.” (World café, Community and sector expert).

Participants also emphasized that almost every job now involves technology, even in traditionally non-tech roles. One participant stated, “my first job was using a POS [Point of Sale] system” (World café, YPWD) and that being able to “understand” and “navigate” that technology was a requirement of the role. This sentiment was reinforced by a specific example from a participant's experience working at a concert venue: “There's no more paper tickets… as an employee … you need to be able to engage with technology to be able to allow access for fans that are coming into the facility.” (World café, YPWD) These are just two examples that highlight how even entry-level positions require digital fluency.

For YPWD the requirement to understand and navigate various types of technologies can present significant challenges. As stated previously, being able to engage with various accessibility features, if there are any at all, can be challenging. But in addition to that, participants reflected an underlying assumption that all young people inherently have digital skills and understand technology. Participants reflected that YPWD have frequently experienced difficulty transitioning from using Google products in a school environment to Microsoft products in the workplace. Assumptions around a built-in baseline digital proficiency for youth results in a lack of intentional interventions designed to teach individuals the foundations needed to engage in an increasingly digital world. Abigail and Jessica's journeys show these assumptions in that they both are seeking to develop basic digital skills to support themselves in seeking employment and building a stable life.

Finally, building digital skills can also be difficult with the rapid changes in technology. While it was not a specific topic covered in the study, participants felt the need to reflect the increasing use of AI in the pathways because of how rapidly AI is evolving and becoming more common in workplaces. Shannon's journey specifically highlights the difficulty in finding quality training opportunities, where the Canadian education system lags behind and community-based resources may not be quality resources. For YPWD, quality, accessible content is critical to advancing their skills. However, due to the rapidly changing nature of AI, having current, accurate, accessible education for each new development is unlikely, making it difficult for YPWD to rapidly upskill and stay competitive in the workforce.

#### 3.3.4 The shortcomings of formal systems & institutions

Participants often reflected on the shortcomings of formal systems and institutions in Canada that made navigating digital skill development difficult for YPWD. The most prominent discussion was around the current education system not adequately preparing YPWD for the workforce, creating critical skill gaps, like digital skills. Participants reflected that educators often do not receive enough or any training on accessible technology, preventing them from teaching students with disabilities how to fully utilize certain programs and technologies that are commonly taught in schools. One participant reflected that she had to teach herself how to use accessibility features, and then ended up showing her teachers how to engage with the technology. While this is only one example of the barriers within the education system, participants reflected barriers like this one is what can lead YPWD to seek community-based skill development.

Looking beyond the education system, participants reflected that people with disabilities often face the challenge of navigating formal systems, from disability benefits, to complex healthcare pathways. The need to navigate a multitude of government and program requirements, bureaucracy, and to consistently self-advocate can leave little room for activities like skill development. These navigation challenges are reflected in Jessica's journey, where participants wanted to capture that Jessica only feels able and motivated to start pursuing her educational goals as a result of obtaining stable housing. Due to her lack of resources, Jessica faces barriers in trying to access healthcare, technology, and other supports that only prolong her reaching her goals and make her journey more challenging.

Participants also reflected a lack of centralization in what community resources and programs are available is a shortcoming of social systems, creating barriers to access. When looking for learning opportunities there was rarely a consistent location participants knew to look. This also often resulted in participants finding courses that were not designed with accessibility in mind. In some cases, disability specific organizations can centralize some of this information and become referral sites, but not every type of disability will have a respective organization.

“I'm aware that not every disability probably has this - but going through your respective NGO bodies. In my case it's CNIB [Canadian National Institute for the Blind]. So those NGOs [act] like an interface letting people know [about opportunities]. Like, I wouldn't have known about this project if it wasn't brought to me by staff at CNIB.” (World café, YPWD).

Additionally, not many programs or courses were designed with integrated learning opportunities. Some participants shared stories about enrolling in many different programs simultaneously to try and meet their learning objectives or career goals. Without any sort of centralized database of services and programs, participants often had to undertake the burden of finding appropriate programs or courses from various places. This barrier to access was reflected across the pathways with the need for information-seeking at the beginning of the journeys.

#### 3.3.5 Structural inequities in career progression

The study found that YPWD who are working often face challenges when trying to upskill so they can progress in their careers, highlighting that career progression can be shaped by systemic inequities that go beyond individual performance. For example, participants reflected that a lack of digital skills and technology training in the workplace also exacerbated employment retention and success for YPWD. Fueled by assumptions that young people already have some amount of digital proficiency, there are very few digital skills that get trained for on the job. One participant recalled, “in my first job… I didn't have much tech training, if any at all… they never really gave me training… they threw you in there thinking you could go with the flow.” (World café, YPWD).

Gaps in employers' knowledge can create barriers to career progression, especially where technology is involved. Participants reflected that for YPWD to continue to develop, or even demonstrate, their digital skills, employers need to understand accessible technologies or at least be open to learning more about them.

“I've been in interview situations where the person on the other side did not understand what a screen reader is. You can kind of sense where they are at with that. If they're interested, they want to know more, they want you to show them. Some just want to shut you down and go “oh our information is private.” (World café, YPWD).

Fear of discrimination and stigma was also shared as a barrier to YPWD upskilling in their careers. Participants shared that YPWD may be afraid to ask for accommodations that would benefit their skill development because of past negative experiences. For example, one participant described an interaction with an employment agency where they were told “I'm too disabled to work…They said I'm too disabled to work and told me to get my healthcare stuff figured out. And try again next year.” (World café, YPWD) Negative experiences like this may create reluctance to disclose accommodation needs that could support equitable participation in the recruitment and employment processes. In the journeys, Khalid's concern about employer bias during his job search exemplifies this challenge.

### 3.4 Opportunities & ‘gain points'

While overall featuring less prominently throughout the data, there were some notable themes surrounding what is helping YPWD on their journeys of developing digital skills and gaining employment.

#### 3.4.1 Connection to resources

While participants reported that the lack of centralization was a challenge when navigating community-based skill development pathways, they also shared that when a person is connected to the right organization or program it can make it easier to access resources. Participants reflected their experiences in Evan's journey, where when he acquires a brain injury from an accident at work, his employer connects him to a doctor to assess which of his skills and abilities have been altered due to the injury. This is helpful because knowing what types of things he is able or unable to do informs what new jobs he may be interested in.

As illustrated, the process of finding and accessing the right service or product for one's needs, whether that be a healthcare service, community program, or something else, can be very challenging and time-consuming for YPWD. Hence, connections between related services and resources that can minimize responsibility on the individual to navigate service access is beneficial.

#### 3.4.2 Community as a resource

Perhaps the most prominent theme amongst the journeys is the presence of community. Whether it is in-person or online, formal and informal communities are essential sources of information, resources, and support. Across the journeys, YPWD relied on their communities to support their needs. Communities acted to fill the gaps of what formal systems lacked. For example, community organizations that offer digital skills education were utilized in six of the journey maps. Participants reflected that skill development often came first from friends and family, and then from accessing community organizations. One participant shared:

“I'm kind of doing that with March of Dimes [Canada]. I just finished the entrepreneurship LinkedIn pathway and now I'm doing one of the digital skill ones that covers Microsoft office, all those platforms.” (World café, YPWD)

Community also provides opportunities for informal support, for example; online forums are spaces where people can receive informal emotional support from others who have gone through similar experiences. These types of communities are also helpful in practical ways, where people can get information, advice, and feedback about a particular skill they are working on developing. For instance, in Khalid's journey he connects with an online community of people with similar interests and experiences to get advice on how he should go about learning a new graphic design skill in an accessible way. Overall, communities offer a wealth of resources. They provide YPWD with the essential support they need in pursuing their educational and professional goals.

#### 3.4.3 Free online resources

As demonstrated across multiple findings, financial barriers to digital skill development can greatly limit YPWD, from purchasing devices or accessible programs, to paying for programs that teach advanced digital skills. However, even with these barriers it was clear that free online resources found on sites like YouTube or online forums like Reddit can greatly benefit YPWD building their digital skills. Participants often reflected that free online resources were low barrier options for skilling and upskilling. This was illustrated in multiple journeys where searching the internet was an initial or key step to improving digital skills.

There are still barriers that can prevent YPWD from accessing free resources online, such as not having accessible technology, or even access to the internet. Even so, participants made it clear that the abundance of information and resources available online for free could greatly diminish barriers to developing digital skills for YPWD.

## 4 Discussion

Engaging community members throughout our study led to a deep understanding of the complexities young people with disabilities face when looking to develop or improve their digital skills. The seven pathways we developed highlighted that many journeys start with seeking information, require YPWD to navigate and foster growth while managing their disabilities, and advocate for themselves, all while coping with emotional stress. Digging deeper, we identified several challenges throughout the pathways including costs associated with community-based skill development, the lack of focus on accessibility, rapidly changing technology, the shortcomings of formal systems, and structural inequities in career progression. Last, we were able to identify some of the strengths when YPWD aim to develop their skills such as the advantages gained when linked with community organizations, the importance of community, and the ability to access free online resources.

Once we had identified the complexities of digital skill development we completed multiple consultations with community members, including YPWD, subject matter experts, community organizations, and service providers to develop recommendations and action plans that can improve skill development pathways for YPWD. Keeping with the principles of community-based research this discussion will focus on the recommendations and actions we heard from our communities on how to improve these pathways. It is important to note that the recommendations discussed below are mostly focused on the federal policy levers that are needed to create systemic change to increase employment opportunities for YPWD, which will ultimately support improved life outcomes. We chose to focus in this area because federal leadership and collaboration with provincial and territorial governments are essential to advancing consistency, equity, and accessibility across Canada, particularly where fragmented systems, uneven standards, and jurisdictional gaps undermine outcomes for YPWD. However, we have also included organizational changes employers can make, and programmatic changes for community organizations that can immediately improve skills development and employment experiences for YPWD.

### 4.1 Improving formal supports to support skill development

First, the findings clearly demonstrated that young people with disabilities face many systemic, financial, health, and service-navigation related barriers that can undermine their ability to successfully enter education, employment, and independent living. In Canada, income and social support programs currently exist to address some of these barriers. However, these programs are often fragmented, inconsistent, and insufficient in addressing the true cost of disability. These supports are largely provided by provincial governments across Canada with some support from the federal government, meaning supports are inconsistent across the country, overly complex, and at times penalizing towards people with disabilities hoping to improve their circumstances (43, 44).

Through the consultations, community members suggested that a coordinated national approach is needed to ensure that YPWD have stable financial resources, housing, reliable mental-health supports, and clear pathways for navigating complex systems. Increasing disability income supports to account for the significantly higher cost of living associated with having a disability is needed to ensure YPWD have the support they need to manage their disabilities while developing their digital skills. Evidence shows the additional cost of having a disability can often create financial stress (44–47) limiting a person's ability to afford non-essential costs such as education and skill attainment. Difficulty navigating complex systems is well known to create additional barriers for people with disabilities (48, 49), meaning better financial support done in combination with providing navigation and coordination support in multiple formats is essential in setting YPWD up for success. In Canada, individuals should be able to easily understand, access, and combine income supports, services, and employment programs without penalty. Christianson-Barker et al. (49) conducted a scoping review examining navigation supports for people with intellectual disabilities and found that navigation supports are needed in many countries, noting that Canada has a particularly fragmented system. They also found there are benefits when navigation supports are put in place for people with intellectual disabilities and their families, such as reduced wait times, collaboration between providers, and more informed decision making. Setting up coordinated systems at the same time as providing proper financial supports is the critical first step in ensuring YPWD have more opportunities for things like digital skill development, which will make them more competitive in the labor market when seeking employment.

Another approach that can support YPWD is to extend the duration, eligibility, and flexibility of support programs to accommodate diverse life paths, ensuring that “late bloomers” in employment, education, or skill development have continuing access to individualized support throughout adulthood. This can include extending the age range of youth or transition-age supports significantly past secondary school completion. Institutions in Canada need to recognize that “aging out” of youth systems often coincides with a time when individuals need increased support and stability in order to pursue their professional or educational goals. Toulany et al. (50) reflect on this, sharing that properly supporting healthcare transitions is critical for young people because they tend to be less focused on their health at this stage of life. Not having the proper support may lead to worse health outcomes for YPWD and ultimately negatively impact their professional or educational goals. Another way to improve support systems is to establish formal requirements for transitional “handoffs” when moving from youth to adult systems, which can better prepare YPWD when seeking employment (51). This includes ensuring that the individualized care common in youth programming is not lost during the transition to adulthood. It is well documented that the transition to adulthood can be a drop off point for people with disabilities and is often a time of heightened stress and uncertainty for young people and their families (52, 53). Community organizations do provide programs to support this transition but our study uncovered that even these programs can have barriers. To improve access to these programs, organizations should consider reducing condition specific inclusion criteria, and take into account the diverse life paths for YPWD when considering program entry points. These changes could allow more YPWD to get the support they need as they transition into adulthood and help prepare them for employment.

### 4.2 Shaping educational supports to increase digital literacy

Consistent with the literature (23, 54), digital literacy emerged from the findings as a key educational gap for many young people with disabilities. Young people with disabilities face barriers in accessing digital education, technology training, and experiential learning opportunities that are universally designed and accessible (23, 54). To help ensure YPWD can achieve full participation in a rapidly evolving digital world there is a need to establish clear standards for digital education throughout education systems in Canada. This includes integrating accessible digital literacy education throughout elementary and secondary schooling. Ensuring that YPWD have equal and equitable access to technological and digital training has been an ongoing challenge in Canada (24). One way to support better access to digital literacy education in elementary and secondary schooling is for teachers, educational aids, and other educational professionals to receive the professional development required to deliver inclusive and accessible digital learning. Our pathways showed how accessibility is often a secondary consideration, and one way that shows up is when educators do not know how to use accessible technology, which can limit their ability to support students with disabilities when learning digital skills. Khanlou et al. (23) stated that even with increasing use of technology amongst young people with intellectual disabilities, insufficient training on accessible technology for educators and parents remains a barrier to digital literacy. Creating a strong foundation in digital literacy requires it to be integrated into children's education, but also ensuring educators have the skills needed to support their students with disabilities.

Beyond the elementary and secondary education systems, supporting digital skill development for YPWD includes creating sustainable funding for community-based digital literacy programs. Our study found that when YPWD have access to community organizations, being connected to programs and supports becomes easier. However, it is common in Canada for community-based programs to be underfunded or to be developed without sustainable funding models in place (55). This can lead to inconsistent access for YPWD and ultimately limit opportunities for skill attainment. Creating more sustainable funding models for community-based programs is necessary to ensure consistently accessible opportunities for digital skill development. This is particularly true for YPWD who may have exited the education system and are looking to continue to build their skills.

For YPWD who pursue post-secondary education, increasing accessible work-integrated learning opportunities can give students the opportunity to develop and refine their digital skills in real-world scenarios. These types of opportunities can increase YPWD's chances of successful employment once they graduate, and can further the Canadian government and industries interest in developing a more diverse Canadian workforce (7). Therefore, developing stronger partnerships between post-secondary institutions and industry can benefit Canadian businesses, while also providing more opportunities for YPWD to grow and refine their skills.

Building on the need to improve education systems, coordinated interventions that address both access to technology and the skills needed to use it effectively is critical for ensuring that YPWD can meaningfully participate in a rapidly evolving digital society. These types of interventions have been developed in community organizations and research has shown they can positively support YPWD in developing digital skills for life and work (27–30). Yet, even with these interventions, significant gaps persist in the affordability, discoverability, and usability of accessible technologies. The pathways we developed demonstrated how these gaps can show up for young people depending on their circumstances, education level, and their disability. The literature further supports this finding in that people with disabilities have less access to technology (24) and often have educators who are not trained in accessible technology (23). Addressing these barriers is not straightforward, but our consultations suggested that community-embedded supports, such as accessible technology lending libraries, or creating a national database of accommodations and assistive tools could help YPWD more easily find and participate in interventions that will build their digital skills. These suggestions from community members would require collaboration from a variety of organizations, institutions and policymakers.

### 4.3 Continuing to advocate for inclusive workplaces

It is well known that creating inclusive workplaces is a key approach to addressing the barriers people with disabilities face when seeking employment. The experiences our participants shared revealed that workplaces in Canada are not overly inclusive, with employers who often lack training in disability inclusion, do not understand accessibility requirements, or lack internal expertise. This can limit YPWD's ability to sustain employment, since they require supportive, knowledgeable, and accessible workplaces. In 2024, the Canadian federal government released a national Employment Standard to work towards more inclusive workplaces. However, this standard is only mandatory for federal offices and organizations. Legislation in Canada that is enforceable across all workplaces, such as Ontario's Accessibility for Ontarians with Disabilities Act require basic accessibility supports and often fall short of the supports people with disabilities actually need (44). Federal or universal legislation could be effective in creating more inclusive workplaces. During our consultations, community members stated that aligning requirements for all employers to the national Employment Standard alongside existing provincial employment accessibility standards is a key next step to building capacity, reducing barriers, and fostering inclusive organizational cultures.

Building on this, our study explored YPWD experiences related to both gaining and sustaining employment. In the workplace, there are many variables that may lead to employees with disabilities struggling to perform their role, not advancing professionally, or even losing their job. Although it is important to help YPWD secure employment, it is equally as important to ensure young people with disabilities are successful through their tenure. As discussed, creating more inclusive workplaces via nationally established standards is one way to ensure continued support throughout the career of a person with disabilities. However, implementing standards can take time, so employers and human resource professionals can take immediate steps to create more inclusive workplaces for YPWD. One key action is training managers on confidently creating inclusive, accessible onboarding and professional development plans for employees with disabilities. When managers and human resource professionals understand accessibility needs in skill development and actively support employees in navigating barriers, YPWD are better able to develop and refine their digital skills. This approach also ensures employers are not assuming YPWD already have digital skills, which our study found could be a barrier to employment.

In addition to this, it is important for YPWD to understand their legal rights regarding accessibility, the duty to accommodate, and protection from wrongful termination. Research has shown that interventions teaching self-advocacy can ensure that people with disabilities understand their rights and can positively impact their lives (56–58). Creating interventions that support self-advocacy and career-navigation training may ensure that YPWD can successfully advance in their careers. This is an area where community organizations can better support YPWD. Re-assessing current programs to add or strengthen training in self-advocacy, including helping YPWD to better understand their legal rights, could help YPWD more confidently navigate the hiring process and workplace. Access to these types of interventions will also help YPWD be able to advocate for continued digital skill development as technology advances. This will become increasingly important as AI continues to change the workplace and modify roles. YPWD who can advocate for themselves will be able to develop their technical and AI literacy skills, remaining competitive in an evolving job market.

### 4.4 Limitations

The primary limitation to our study was the relatively small sample size, with only 22 participants included across the study. This was somewhat mitigated through our regular discussions with the three stakeholder groups we engaged to support our community-based research approach. We are confident that the iterative analysis that built on findings from various study activities, along with consulting these groups at regular intervals, resulted in rigorous findings. However, more participants could have led to greater depth to the findings.

Our small sample size also limited geographical representation in our study. We aimed to recruit participants from across Canada, but were unable to gain representation from all provinces, indigenous communities, rural areas, and no participants from Canada's territories were included. This limitation means these experiences and perspectives were not captured. To mitigate this limitation the community and sector experts could offer some insight in these areas. Also, our incorporation of academic literature, community evidence and population data in the pathway development and resulting recommendations also helped us capture some of the unique considerations in communities across Canada. It is important to note this is an ongoing challenge for research in Canada; the geographical size and variation of the country often make it difficult to development interventions and recommendations that are applicable across the country.

## 5 Conclusion

Conducting research with lived experience and academic communities is invaluable when unpacking complex and nuanced issues like digital skill development for YPWD. Our study demonstrated that even when focusing on developing a skill area there are a multitude of factors that people with disabilities, and especially YPWD, have to consider and work through. Being able to engage with community members with a range of experiences and knowledge helped us to create pathways that clearly showed the commonalities YPWD face, the challenges along these pathways, as well as where there are opportunities to support digital skill development and reduce barriers for YPWD. Overall, creating accessible and inclusive community-based digital skill development pathways requires systemic change in Canada that is driven by the federal government, encourages collaboration across systems and organizations, and remains grounded in the lived experiences of young people with disabilities. In summation, from our study, community members recommended that:

1. There needs to be strengthened income, mental health, housing, and navigation supports for young people with disabilities in Canada.
2. There needs to be clear and accessible digital literacy education that is embedded in education systems starting at a young age through to post-secondary education.
3. Enhanced access to technology and accessible technologies for young people with disabilities can ensure they equitably develop their digital skills for life and employment.
4. Clear standards and updated legislation are needed to create universally accessible and inclusive workplaces.
5. Expanding employment and education supports, such as self-advocacy and career-navigation training, can ensure role success and career advancement.
6. Creating new, or improving current programs in community organizations that have less restrictive inclusion criteria and take into consideration the diverse life paths of people with disabilities.

Continued research exploring how to best implement and action the recommendations our community members put forward is also needed. Research that explores the specific digital skill development and employment needs of YPWD living in indigenous, rural and remote communities would help to further contextualize our findings across Canada. Ensuring solutions are grounded both in evidence and community insights from across Canada can potentially lead to interventions and systems that successfully support young people with disabilities in attaining the skills they need to live thriving and full lives.

## Data Availability

The raw data supporting the conclusions of this article will be made available by the authors, without undue reservation.
